# *Ruta Angustifolia* Essential Oil: Behavioral and Insecticidal Effects Against Larvae and Adults of *Tribolium castaneum*

**DOI:** 10.1002/cbdv.202402043

**Published:** 2024-11-27

**Authors:** Houssam Annaz, Hamass Zerrad, Mouna Moullamri, Ayoub Ajaha, Ayoub Kounnoun, Francesco Cacciola, Ammar B. Altemimi, Roberto Laganà Vinci, Abdelhay Arakrak, Amin Laglaoui, Noureddin Bouayad, Kacem Rharrabe

**Affiliations:** ^1^ Research team Agricultural and Aquaculture Engineering FPL Abdelmalek Essaadi University Tetouan Morocco; ^2^ Research team Biotechnologies and Biomolecular Engineering Faculty of Science and Technology - Abdelmalek Essaadi University Tangier Morocco; ^3^ Research Laboratory Biology, Environment and Sustainable Development ENS. Abdelmalek Essaadi University Tetouan Morocco; ^4^ Regional Analysis and Research Laboratory National Office of Food Safety ONSSA Tangier Morocco; ^5^ Messina Institute of Technology c/o Department of Chemical Biological, Pharmaceutical and Environmental Sciences, f ormer Veterinary School University of Messina, Viale G. Palatucci snc 98168 Messina Italy; ^6^ Department of Food Sciences College of Agriculture University of Basrah Iraq

**Keywords:** Feeding deterrent, Direct contact, Repellent, Feeding preference, Stored grain pest

## Abstract

The study aims to evaluate the behavioral and insecticidal effects of *Ruta angustifolia* EO (RAEO) against larvae and adults of a major pest insect, *Tribolium castaneum*. RAEO exhibited a strong repellency against both adults and larvae where percentages as high as 74 and 72 % of PR (Percentage of Repellency) were recorded respectively, at 0.47 μL/cm^2^ after 2 h. Regarding the antifeedant bioassay, no feeding deterrence was recorded in adults while an increase in appetite was registered in larvae exhibiting −80 % of FDI recorded at 0.2 μL/pellet. In terms of feeding preference, the response significantly depended on the developmental stage (F=97.19, P<0.05) and larvae were attracted to treated pellets recording percentages of 84 and 73 % of PI (Preference index) after 5 and 30 min at 0.05 μL/pellet; on the other hand, adults expressed a repulsive behavior at 0.2 μL/pellet with a PI of −81.1 (20 min), and −69.2 % (30 min). Regarding topical application toxicity, a significant difference was observed between larvae and adults (p<0.05 F=174.56). The study promotes the use of RAEO as a repellent and bioinsecticide for the control of larvae and adults of *T. castaneum*.

## Graphical abstract

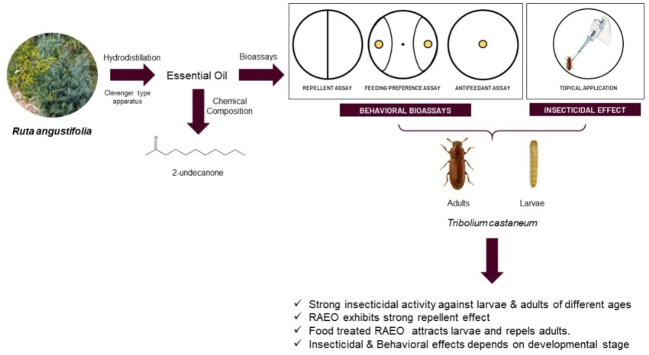

## Introduction

Pest Insects are considered one of the most detrimental pests that can cause significant damage to stored products in milling, processing facilities, commercial warehouses, and retail stores.^[1]^ These pests are considered a significant risk to food safety, especially with the alarm side effects of climate change and the increase of global warming which can create optimal conditions for their proliferation.^[2]^ The red flour beetle *Tribolium castaneum (T. castaneum)*, is considered one of the commonly found pest insects and is known for infesting a wide range of stored food commodities of economic importance.^[3]^

To get rid of this species, management strategies often include the use of conventional chemical insecticides as the primary solution.[^4^, ^5^, ^6^] However, The overuse of these chemicals has led to the continuous appearance of resistance cases in this pest's populations^[7]^ Moreover, these chemicals pose a threat to the environment as they persist in soil and infiltrate into groundwaters leading to detrimental consequences associated with soil and water contamination which impacts human health, soil biodiversity, and viability of non‐target organisms.[^8^, ^9^] Consequently, it is crucial to develop new alternatives to replace or diminish the reliability of these chemicals.

In this regard, botanicals or natural products emerge as promising eco‐friendly alternatives for the control of this pest. Plants are considered sustainable production engines of natural molecules which attracted researchers all over the world to evaluate their activities against different pests including *T. castaneum*.[^10^, ^11^] These plants, especially medicinal and aromatic plants (MAPs), are widely distributed across the world gathering large numbers of species containing a mine of molecules that could be the next potential insecticide.[^12^, ^13^]

For instance, the *Ruta* genus, which belongs to the Rutaceae family, comprises over 40 species majorly found in the Mediterranean region.^[14]^ The phytochemical studies conducted on these species highlight the presence of amino acids, saponins,^[15]^ alkaloids, flavonoids, coumarins, tannins, volatile oil, glycosides, sterols and triterpenes.^[16]^ They are used in the traditional medicine of many countries for the treatment of a variety of diseases. Exciting, diaphoretic, antiseptic, antispasmodic, anthelmintic emmenagogue, abortifacient and anti‐inflammatory properties are inferred to *R. chalepensis var. bracteosa, R. graveolens* and *R. angustifolia. R. tuberculata* treats bone and joint pain, dysmenorrhea, infertility in women, anemia and headache.^[17]^

Moreover, essential oils (EOs) of the *Ruta* genus were reported in the literature to exhibit antimicrobial effects against different bacterial and fungal strains.^[18]^ Besides, These oils also exhibited antiparasitic and insecticidal activities which promote the use of *Ruta* species as a potential alternative for commonly used synthetic chemicals.[^19^, ^20^]

**Figure 1:**
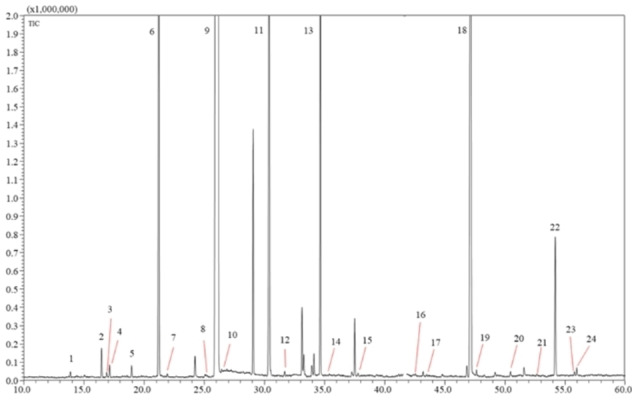
GC‐MS analysis of the *Ruta angustifolia* essential oil. For peak identification see ref. [22]..

**Figure 2:**
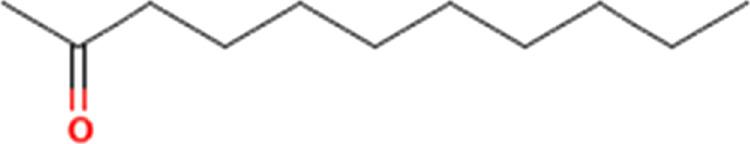
2‐undecanone, *Ruta angustifolia* essential oil major compound

However, not all *Ruta* species were fairly evaluated. According to a recent review, *Ruta chalepensis* L., *Ruta graveolens* L., and *Ruta montana* L. were the three most extensively studied species.^[21]^ On the other hand, *Ruta angustifolia* EOs have received limited research attention regarding their chemical composition and biological activity. In this regard, in our previous study,^[22]^ we reported the chemical characterization of such a species along with antioxidant and antimicrobial activities, the latter demonstrated against numerous bacterial and fungal strains. The chemical composition of its EO was dominated by 2‐undecanone (nonyl methyl ketone) (Figure 1) with a percentage of 80.96 %, followed by Moskachane B (4.43 %), Undecyl methyl ketone (2.26 %) and Octyl methyl ketone (2.02 %). In total, ketones dominate the volatile content of *R. angustifolia* EO accounting for 87.4 of the entire volative content. Figure 2 reports the structure of 2‐undecanone.

Results are similar to a previous study reporting the chemical composition and antimicrobial activity of the essential oils from four *Ruta* species growing in Algeria.^[23]^ In terms of the insecticidal, antifeedant, and repellent effect of this EO, to date, no reports have addressed this application either against pest insects in general, or *T. castaneum* in particular.

Hence, this study aims to evaluate the behavioral and insecticidal activities of *Ruta angustifolia* essential oil (RAEO) against larvae and adults of a major stored product pest, *T. castaneum*. The behavioral effects evaluated consisted of the repellent, antifeedant, and feeding preference effect of RAEO while the insecticidal effect was evaluated against larvae different exposure routes to the essential oil, starting from repellent effect to antifeedant and feeding preference bioassay, and finishing with topical application toxicity bioassay. These effects were assessed comparatively against both larvae and adults of this pest insect. This study will provide evidence of the insecticidal/behavioral effect of this *Ruta* species for the first time to promote its use in the management of stored product pests.

## Results and Discussion

### Topical Application Toxicity

As expressed in Table 1, RAEO exhibited a strong toxic effect against *T. castaneum* especially at the highest dose of 0.08 μL/insect. Mortality was significantly influenced by developmental stage (p<0.05 F=174.56). For instance, 5^th^ instar larvae turned out to be the least sensitive (8 % of mortality was recorded at the highest dose after 24 h), compared to the adults one that were the most sensitive one. On the other hand, 1‐day‐old adults were the most sensitive while 7‐day‐old were less sensitive where 100 and 58 % of mortality were recorded respectively after 24 h.

**Table 1:** Topical application toxicity of *Ruta angustifolia* essential oil against Larvae (L5) and Adults (A1, A7, A14, A21) of *Tribolium castaneum*.

	Concentration (μL/insect)								
	0.02			0.04			0.08		
	24 h	48 h	72 h	24 h	48 h	72 h	24 h	48 h	72 h
L5	4±2a	8 ±2a	14±2a	6 ±2a	8 ±2a	12±2a	8 ±2a	30±3a	58±2a
A1	10±0a	12 ±2a	16±2a	38 ±4	40±4b	40±4b	100±0b	100±0b	100±0b
A7	2±2a	4 ±2a	4 ±2b	10±3a	10±3a	10±3a	58 ±2c	64±2c	64±2c
A14	6±2a	10 ±3a	12 ±2b	42±4b	44±2b	48±4b	70 ±3d	70±3c	72±2c
A21	8±2a	8 ±2a	8 ±2b	40±3b	48±2b	48±2b	68 ±4cd	70±3c	70±3c
F	2.5	1.57	4.46	28.82	42.73	35.08	174.563	86.278	54.417
*P*	0.075	0.221	0.01	<0.05	<0.05	<0.05	<0.05	<0.05	<0.05

L5=5th instar Larvae, A1 =1 day old adults, A7=7 day old adults, A14=14 days old adults, A21=21 days old adults. Values expressed as the mean of mortality percentage ± Standard error for five replicates. Results are considered significant when the letters are different at a P <0.05 at different developmental stages using One‐way ANOVA and Tukey post hoc

This toxicity investigation for RAEO is hereby reported for the first time and can be attributed to its major compound, *viz*. 2‐undecanone. The latter was exclusively studied in house fly *Musca domestica* exhibiting significant topical application toxicity with an LD50 value of 58.1 μg/fly.^[24]^ Additionally, 2‐undecanone induced 100 % mortality at 1 % against three stored product pests (*Tenebrio molitor, Tribolium confusum*, and *Acanthoscelides obtectus)* in filter paper contact bioassay.^[25]^ When applied to grains, 2‐undecanone caused 98.9 % and 87.8 % of mortality in *Tenebrio molitor* adults and larvae respectively after 7 days at 1000 μL/kg wheat.^[26]^ As for sealed petri dish assays, 2‐undecanone exhibited strong fumigant toxicity ranging between 94 and 97.9 % at 2.8 μg/cm^3^ against three species of flies, namely house fly, flesh fly: and blow fly, respectively, after 2 h.^[23]^ On the other hand, it is important to mention that other 2‐undecanone‐rich EOs were reported in the literature for their insecticidal effect. For instance, *Ruta chalepensis* (64.35 % of 2‐undecanone) caused 100 % mortality in *T. castaneum adults after* 48 hours of exposure at 0.62 μL/mL.^[27]^ Moreover, *R. graveolens* EO (16.22 % of 2‐undecanone) demonstrated strong toxicity against *Ephestia kuehniella* and *Ectomyelois ceratoniae* leading to an (LC50) values of 1.02 μL/L air and 1.97 μL/L air respectively.^[28]^ The low sensitivity of larvae to RAEO observed in our study, complies with the studies reported in refs.[^29^, ^30^] carried out on *T. castaneum*, demonstrating reduced sensitivity to essential oils through contact and fumigant toxicity. Additionally, similar results were reported in *Trogoderma granarium and Cryptolestes ferrugineus* larvae and adults. ^[26,31]^ However, exceptions exist, as demonstrated by Plata‐Rueda et al.^[32]^ who reported increased sensitivity in *Tenebrio molitor* larvae compared to adults. These findings imply the existence of various factors influencing this activity. Factors such as the nature of the essential oil and the physiological differences between larvae and adults play pivotal roles. Consequently, it is crucial to carry out insecticidal bioassays on both developmental stages to determine their sensitivity and the efficiency of the EO.

### Antifeedant Effect

*T. castaneum* adults and larvae did not express different antifeedant behavior towards pellets treated with RAEO *(*Table 2 *)*. In terms of adults, no disruption was observed in the four nutritional indexes while larvae's appetite increased especially at 0.2 μL/pellet leading to −80 % of FDI. Despite the lack of an antifeedant effect on nutritional indexes was observed, statistical analysis revealed that there was a significant difference between larvae and adults in their response to RAEO in all four nutritional indexes (p<0.05 and F equals 4.9, 8.5, 5.9, and 4.5 respectively for RGR, RCR, ECI, and FDI).

**Table 2:** Antifeedant effect of *Ruta angustifolia* essential oils on Adults and Larvae of *Tribolium castaneum*.

	Concentration (μL/pellet)	RGR^[]^ (mg/mg/day)	RCR^[*]^ (%)	ECI^[*]^ (%)	FDI[^*^] (%)
Adults	**0**	0,023 ±0,002^**[a]**^	0,204 ±0,014^**[a]**^	11,2 ±1,1 ^**[a]**^	0,000 ^**[a]**^
	**0,05**	0,027 ±0,010^**[a]**^	0,196 ±0,007^**[a]**^	13,9 ±5,3 ^**[a]**^	0,39 ±6,3^**[a]**^
	**0.1**	0,025 ±0,004^**[a]**^	0,235 ±0,010^**[a]**^	10,6 ±1,8^**[a]**^	−19,7 ±6,4^**[a]**^
	**0.2**	0,023 ±0,002^**[a]**^	0,217 ±0,014^**[a]**^	10,8 ±1,6^**[a]**^	−7,5 ±4,3 ^**[a]**^
Larvae	**0**	‐0,010 ±0,005^**[a]**^	0,079 ±0,017^**[a]**^	−34,6 ±27,2^**[a]**^	0,000^**[a]**^
	**0,05**	0,006 ±0,010^**[a]**^	0,114 ±0,012^**[a]**^	1,64 ±11,7^**[a]**^	−13,0 ±13,5^**[a]**^
	**0.1**	0,011 ±0,029^**[a]**^	0,213 ±0,065^**[a]**^	−1,5±11,6^**[a]**^	−21,4 ±11,3^**[a]**^
	**0.2**	0,007 ±0,017^**[a]**^	0,208 ±0,039^**[a]**^	−9,1 ±17,9^**[a]**^	−80,9 ±27,7^**[a]**^

[a] Values expressed as the mean of mortality percentage ± Standard error for five replicates. Results are considered significant when the letters are different at a P <0.05 at different concentrations using One‐way ANOVA and Tukey post‐hoc.*RGR : Relative Growth Rate *RCR : Relative Consumption Rate *ECI : Efficacy of Conversion of Ingested Food *FDI : Feeding Deterrence Index

Overall, this effect was evaluated for the first time using this EO In the literature, no antifeedant effect was reported using RAEO or any other *Ruta* species with the exception of one study where the antifeedant effect against *Spodoptera frutiperda* larvae was explored.^[33].^

### Feeding Preference

**Figure 3:**
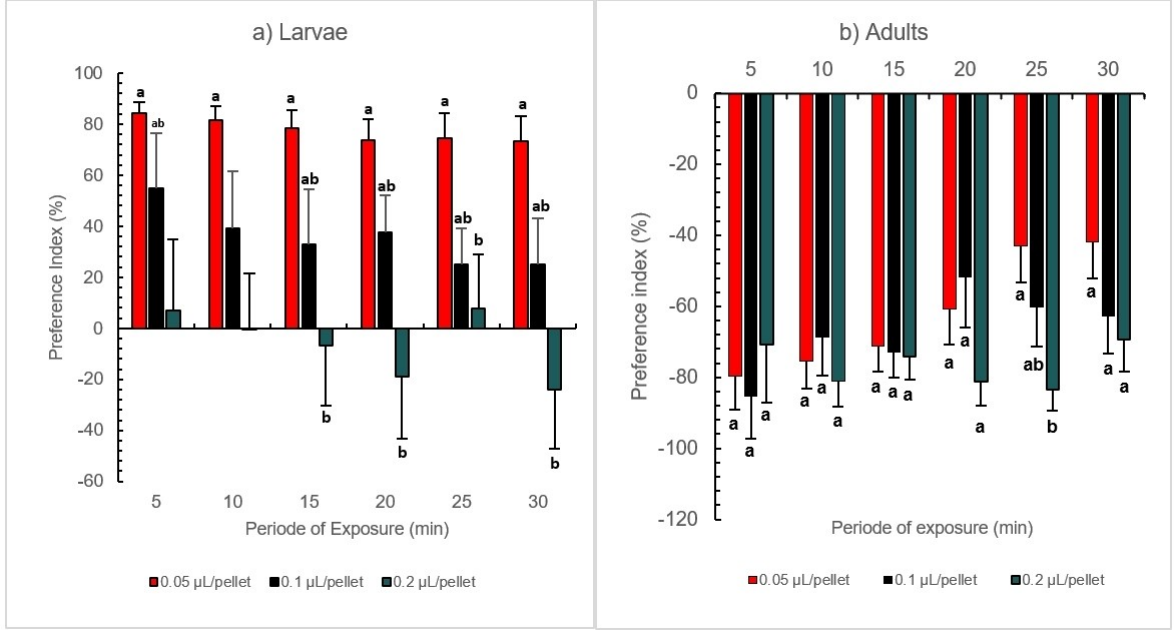
Feeding Preference of (a) larvae and (b) adults of *Tribolium castaneum* exposed to *Ruta angustifolia* essential oil. Values are expressed as the mean of preference index (PI) ± standard error. Significant differences are determined between treatments for ten replicates and means with the same letter are not significantly different following one‐way ANOVA and Tukey post‐hoc.

**Figure 4:**
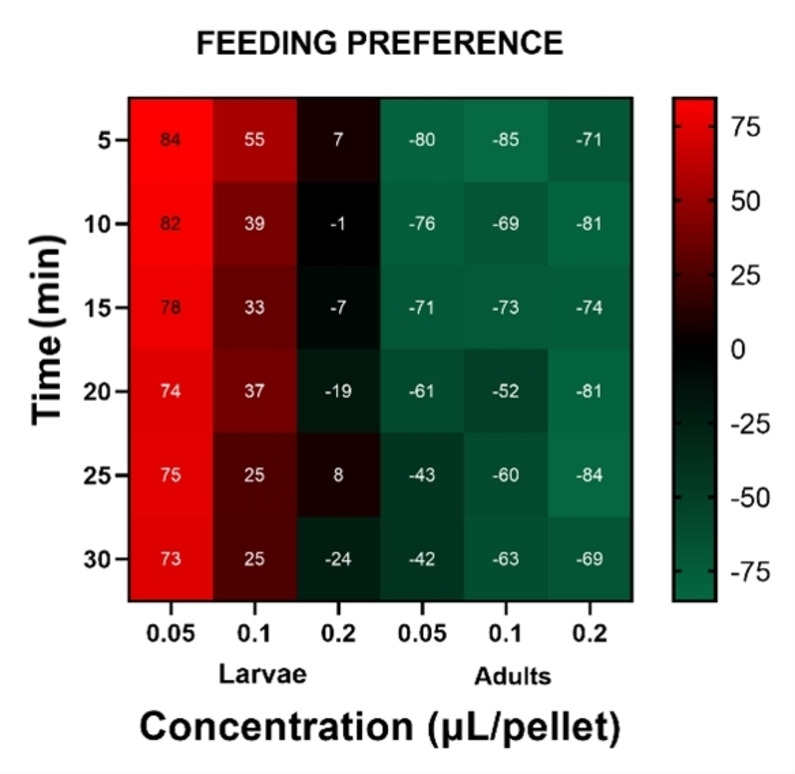
Heatmap representing the feeding behavior of Larvae and Adults of *Tribolium castaneum* to *Ruta angustifolia* EO. Values expressed as the mean of preference Index (%) for ten replicates.

The response of larvae and adults to different concentrations of RAEO depends on developmental stages (Figure 3, 4). For example, larvae were attracted to treated pellets where PI (preference index) of 84 and 73 % were recorded as the highest preference index (PI) after 5 and 30 mins respectively, at the lowest concentration (0.05 μL/pellet). The PI decreased significantly in a dose‐dependent manner reaching 55 and 7 % respectively for 0.1 and 0.2 μL/pellet after 5 minutes and decreasing to 25 and −24 % after 30 min. In contrast, adults expressed a repulsive behavior towards pellets treated with RAEO where the highest value of PI (−85 %) was recorded for 0.1 μL/pellet after 5 minutes. Overall, the highest concentration (0.2 μL/pellet) expressed the optimal response, with −80.9 %, −81.1 %, and −69.2 % recorded after 10, 20, and 30minrespectively. Statistical analysis indicates a highly significant difference between concentrations and also life stages throughout the 30‐minute experiment. For example, there is a highly significant difference between life stages (F=97.2 p<0.05) and concentrations (F=4.74, p<0.05) respectively after 10 minutes of exposure.

This effect is hereby reported for the first time; this adapted bioassay makes it a first of its kind in stored product insects especially since it focuses on feeding behavior, and not mortality or other commonly studied parameters. For instance, in the case of *Trogoderma granarium*, both adults and larvae avoided the food sources, with no significant difference noted. ^[26]^ Similarly, Lazarević et al. ^[34]^ found that both developmental stages of the Colorado potato beetle were repelled by the essential oil pure compounds camphor and thujone, challenging the trend observed in our study. Furthermore, they reported that the choice of treated leaf pellet did not depend on the life stage of the Colorado potato beetle *Leptinotarsa decemlineata*. The observed attractant effect of larvae in our study suggests a promising approach for eliminating larvae from stored products. This difference in responses may be attributed to factors related to the insect's olfactory and gustatory systems, as well as the concentration of the treatment employed. Therefore, our findings open avenues for further exploration and underscore the need for a nuanced understanding of feeding behavior in stored product insects.

### Repellency on Filter Paper

**Figure 5:**
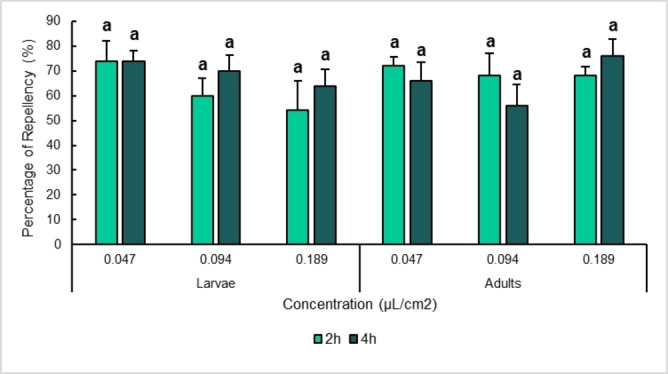
Repellent Effect of *Ruta angustifolia* essential oil against larvae and adults of *Tribolium castaneum*. Values are expressed as mean percentage of repellency (PR) ± standard error. Significant differences are determined between treatments for five replicates and means with the same letter are not significantly different following one‐way ANOVA and Tukey post‐hoc.

**Figure 6:**
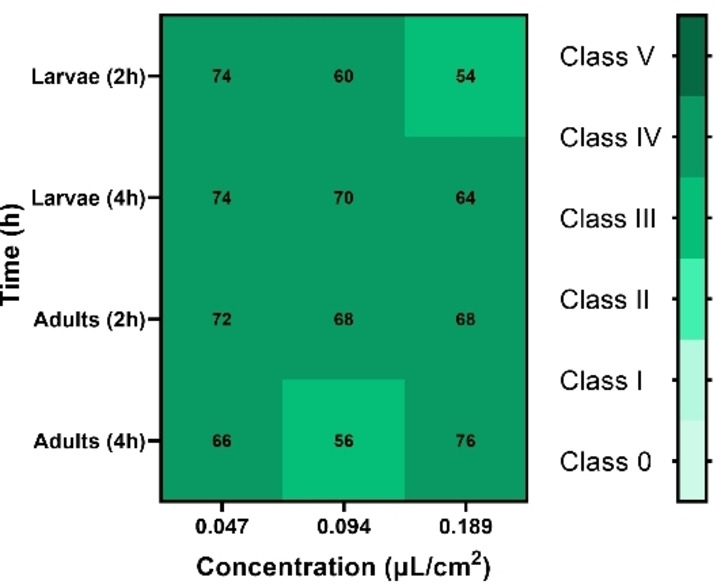
Heat map representing The repellent effect of *Ruta angustifolia EO* against larvae and adults of *Tribolium castaneum*. Values are expressed as the mean percentage of repellency (PR) for five replicates.

RAEO exhibited effective repellent activity against both larvae and adults of *T. castaneum* as illustrated in Figures 5, 6. For the larvae, the highest repellency rate was observed at the lowest concentration tested (0.047 μL/cm^2^), with a PR (percentage of repellency) of 74 after 2 and 4 h. At higher concentrations (Class IV), no significant difference was observed, where 70 and 64 % of PR were recorded at 0.094 and 0.189 μL/cm^2^, respectively, after 4 h. For the adults, the highest PR (76 %) was observed at the highest concentration after 4 h belonging to class IV. The lowest concentration, also, showed high PR of 72 % and 66 % after 2 and 4 h, respectively. Statistical analysis showed no significant difference between concentrations (F=3.130 and p=0.57) or between larvae and adults (F=2.593 and p=0.117).

Nevertheless, no study has reported this effect using RAEO against *T. castaneum*, or any other insect species. This repellent effect is highly due to 2‐undecanone, which has been reported to display a significant repellent effect against some pest insects. For instance, 1 % of 2‐undecanone induced the highest levels of repellency against adults of three stored product pest insects (*Tenebrio molitor, Tribolium confusum*, and *Acanthoscelides obtectus*.^[26]^. Fusková et al.^[35]^ reported a significant repellent effect of this compound on adults of the western corn rootworm *Diabrotica virgifera*. Against flies, In a two‐choice behavioral assay, 194.6 μg/cm^2^ of 2‐undecanone gave 84.7 % and 80.7 % overall mean repellencies, respectively when exposed to blow flies and house flies.^[23]^ Other plants containing this major compound also showed promising levels of repellency; for instance, 1.92×10^7^ ng/cm^2^ of *Ruta chalepensis* EO (40.1 % of 2‐undecanone) showed a potent repellent effect against both male and female of *Aegorhinus superciliosus* using four‐arm olfactometric bioassays.^[36]^ Moreover, *Ruta graveolens* (44.7 % of 2‐undecanone) repelled *Sitophilus zeamais* adults especially when loaded in mineral matrices.^[37]^ Additionally, *Ruta chalepensis* EO (64.35 % of 2‐undecanone) displayed 100 % PR against adults of *T. castaneum* at a dose of 0.038 μL/mL following the same protocol of repellency.^[27]^ As for the absence of significance between larvae and adults, the same observation was reported in *T. castaneum* in ref.^[38]^ The same observation was also reported in other stored product species such as *Tenebrio molitor* ^[32]^ and *Tribolium confusum*.^[39]^

## Conclusions

The present study highlights the effectiveness of RAEO in repelling both larvae and adults of *T. castaneum* which is crucial to protect stored products from their invasion. Our findings reveal a strong significant difference in the feeding behavior of larvae and adults either in the feeding deterrence or feeding preference bioassays. This study also provides compelling evidence of the strong and stage‐dependent insecticidal effect through direct contact. Overall, these results contribute significantly as novel evidence of the potent behavioral and insecticidal effects of this EO, suggesting the necessity of including both feeding stages in further studies, and possible inclusion in formulation or in combination with other insecticides to reduce the reliance on these products.

## Experimental Section

### Insect Culture

Larvae and adult individuals of the red flour beetle (*Tribolium castaneum*) used in this study were obtained from a laboratory culture at the Faculty of Science and Technology in Tangier, Morocco. The beetles were raised in a mixture of wheat flour and dried yeast in a 1 : 19 ratio (w/w), and were kept in bottles at a temperature of 30 °C and humidity of 60±5 % in the dark. Fifth instar larvae were used in all bioassays and experiments, while 7–14 days adults were used for antifeedant, feeding preference, and repellent bioassays. For toxicity by topical application, adults 1, 7, 14, and 21 days old were used and labeled as A1, A7, A14, and A21 respectively.

### Plant Material and Essential oil Extraction

*Ruta angustifolia* plant materials, extraction procedure as well as chemical composition assessment were conducted as stated in^[22]^ In summary, wild *Ruta angustifolia was* harvested from Targuist, Morocco in June 2019. The identity of the plant was confirmed by Pr. Mohammed Bakkali, and a voucher of the specimen (Code MPU008384) was deposited in the Department of Botany and Plant Ecology of the Scientific Institute of Rabat (Morocco). After harvest, the aerial parts were dried in obscurity at room temperature for 10 days. One hundred grams of dried plant material was subjected to hydrodistillation using a Clevenger‐type apparatus for 3 h.

### Chemical Characterization

The chemical composition, of the essential oil of *Ruta angustifolia* was analyzed using gas chromatography‐flame ionization detector/mass spectrometry (GC‐FID/MS) In summary, GC‐MS analyses were carried out on a GCMS‐QP2020 system (Shimadzu Europa GmbH, Duisburg, Germany) equipped with a split‐splitless injector and ‘AOC‐20i’ auto‐injector system. The column was an SLB‐5 ms fused‐silica capillary column (30 m×0.25 mm i.d.×0.25 um df film thickness), which was obtained from Merck Life Science (Merck KGaA, Darmstadt, Germany).

### Topical Application Bioassay

Topical application bioassay, also known as contact toxicity, was carried out by applying different concentrations directly to the body of the insect. Three concentrations were prepared in acetone (2, 4, and 8 %) giving a final concentration of 0.02, 0.04, and 0.08 μL/insect. 1 μL of each concentration was applied to the body of the adult/larva using a precision micropipette (0.5–10 μL) while the control received 1 μl of acetone. Each concentration was performed in five replicates and The mortality rate was recorded after 24, 48, and 72 h. No correction was needed as no mortality was recorded in the control.

### Antifeedant Bioassay

The antifeedant effect of essential oils was carried out using the method proposed by Huang et al.^[40].^ with some modifications. Flour pellets were prepared according to the method by Xie et al.^[41].^ where 10 g of the culture media (1 : 20 g/g) of Yeast and flour was added to 20 mL of distilled water and continuously stirred. Aliquots of 100 μL were then pipetted onto a plastic petri dish. The flour pellet was then left overnight to dry. The next day, dry flour pellets were equilibrated at 30 °C for 24 h, and only the ones weighed from 36 to 39 mg were used.

Each pellet was next treated with 10 μL of each EO concentration (0.5, 1, and 2 %) giving a final concentration of 0.05, 0.1, and 0.2 μL/pellet respectively. The treated flour pellets were then left at room temperature for 20 minutes to ensure the complete evaporation of acetone. The disk was next weighed and placed in a petri dish containing 5 adults/larvae previously weighted and starved for 24 h. Control pellets received acetone only and followed the same procedure. 3 days later, Insects and floor pellets‘ weight were weighed to calculate nutritional indexes following the following refs. [42, 43].⋅

Relative Growth Rate (RGR):

$$\mathrm{RGR}=\frac{\left(A-B\right)}{B\;x\;\mathrm{day}}$$

where: **A**: weight of live insect after the experiment (mg); **B**: weight of insect before the experiment (mg)

Relative Consumption Rate (RCR):

$$\mathrm{RCR}=\frac{D}{B\;x\;\mathrm{day}}$$

where: **D**: weight of food consumed by the insect (mg)

Efficacy of Conversion of Ingested Food (ECI)

$$\mathrm{ECI}=\frac{\mathrm{RGR}}{\mathrm{RCR}}X\;100$$

Feeding Deterrence Index (FDI):

Where: **C**: Consumption of control diet and **T**: Consumption of treated diet.

The experiment was conducted in 5 replicates. For the four concentrations and the control.

### Feeding Preference Bioassay

**Figure 7:**
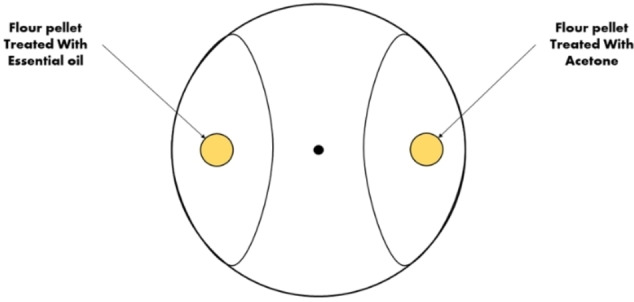
Food Preference Bioassay Setting.

A preference test for food treated with essential oils for Larvae/adults of *T. castaneum* was conducted using flour pellets prepared following^[41]^ and following the protocol by Rharrabe et al.^[44].^ with some modifications as illustrated in Figure 7. Two pellets were separately treated, one with 10 uL of the three concentrations used in the antifeedant test (0.5, 1, and 2 %) giving final concentrations of 0.05, 0.1, and 0.2 μL/pellet and the second with acetone. The two pellets were then left for 20 minutes at room temperature to allow the evaporation of the solvent and then deposited separately in the middle of the two zones. Test and control areas have been marked in each petri dish. The number of individuals in each area was counted every 5 minutes for 30 minutes. The preference index (IP) was calculated from the distribution of individuals in each area using the following formula previously described.

$$\mathrm{IP}=\left[\frac{\mathrm{Nt}-\mathrm{Nc}}{\mathrm{Nt}+\;\mathrm{Nc}}\right]X\;100$$

**N_t_** =Number of larvae/adults observed in the control area,

**N_c_** =Number of larvae/adults observed in the test area.

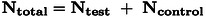




When the PI value is positive, individuals are attracted to the food treated. However, when it is negative, individuals are repelled or express a negative preference for the food treated with the essential oil.

Each treatment was reproduced 10 times using 10 larvae/adults per petri dish. Each individual has been tested only once.

### Repellency Bioassay

The repellent effect was tested according to Jilani et a.^[45]^ Filter paper circles (9 cm) were cut into two halves of which one received 300 μL of each concentration (0.5, 1, and 2 %) dissolved in acetone giving a final concentration of 0,047, 0.094, and 0.189 μL/cm^2^ respectively. While the other half was treated with 300 μL of acetone. Next, treated and control halves were left to dry for 3 minutes and then attached edge to edge with duct tape and placed into a petri dish (9 cm in diameter). Twenty individuals (unsexed adults or larvae) were released in the middle. The number of individuals that settled on each half of the filter paper was counted after 2, and 4 h. The average count was converted to a percentage of repellent (PR) using the following formula:^[46]^

$$\mathrm{PR}=\left[\frac{\mathrm{Nc}-\mathrm{Nt}}{\mathrm{Nc}+\mathrm{Nt}}\right]X\;100$$

**Nc** Number of larvae/adults counted in the control half

**Nt** Number of larvae/adults counted in the treated half

Five replicates were reproduced of each treatment

PR value was used to classify repellency classes from **0** to **V**:

**Class 0**: The percentage of repulsion ≤0.1 %: not repellent;

**Class I**: 0.1 %≤PR ≤20 %: very poorly repulsive;

**Class II**: 20.1 %≤PR ≤40 %: moderate repellent;

**Class III**: 40.1 %≤PR ≤60 %: good repellent;

**Class IV**: 60.1 %≤PR ≤80 %: very repellent;

**Class V**: 80.1 %≤PR≤100 %: perfect repellent

### Statistical Analysis

All statistical analyses were conducted using IBM's software SPSS V25.0All data were analyzed using one‐way ANOVA followed by Tukey Test as post‐hoc at a significance level of p<0.05. Two‐way ANOVA was also assessed to determine the significance between developmental stages and concentrations. Tables of statistical analysis for all bioassays are provided in supplementary materials.

## Author Contributions

**Houssam Annaz**: Methodology, Investigation, Visualization, Data curation, Writing ‐ Original draft ‐ **Hamass Zerrad**: Investigation, Writing ‐ Review & Editing‐ **Mouna Moullamri**: Investigation, Writing ‐ Review & Editing‐ **Ayoub Ajaha**: Writing ‐ Review & Editing,– **Ayoub Kounnoun**: Writing ‐ Review & Editing, ‐ **Francesco Cacciola**: Validation, Writing ‐ Review Editing ‐ **Ammar B. Altemimi**: Writing ‐ Review Editing ‐ **Roberto Laganà Vinci** : Writing ‐ Review Editing‐ **Abdelhay Arakrak**: Validation ‐ Writing ‐ Review Editing‐ **Amin Laglaoui**: Validation ‐ Writing ‐ Review Editing ‐ **Noureddin Bouayad**: Supervision ‐ Writing – Review Editing ‐ **Kacem Rharrabe**: Conceptualization – Project Administration ‐ Validation ‐ Supervision ‐ Writing ‐ Review Editing

## Conflict of Interests

The authors declare no conflict of interest.

1

## Data Availability

The data that support the findings of this study are available from the corresponding author upon reasonable request.

## References

[1] A. R. Gerken , J. F. Campbell , Ann. Entomol. Soc. Am. 2022, 115, 239–252.

[2] C. Adler , C. Athanassiou , M. O. Carvalho , M. Emekci , S. Gvozdenac , D. Hamel , J. Riudavets , V. Stejskal , S. Trdan , P. Trematerra , J Stored Prod. Res. 2022, 97, 101977.

[3] S. Zhang , S. Han , L. Xiong , Y. Hou , X. Gao , X. Tang , Nongye Gongcheng Xuebao/Transactions of the Chinese Society of Agricultural Engineering 2022, 38, 303–309.

[4] R. Khatun , W. Islam , K. A. M. S. H. Mondal , J Biosci. (Rajshari) 2010, 18, 84–87.

[5] F. H. Arthur , J Stored Prod. Res. 2018, 76, 151–160.

[6] N. Subekti, R. Saputri, *AIP Conf. Proc*. **2019**, *2155*.

[7] S. G. Gautam , G. P. Opit , E. Hosoda , J Econ. Entomol. 2016, 109, 2525–2533. 27744283 10.1093/jee/tow221

[8] Y. Geng , J. Ma , R. Zhou , R. Jia , C. Li , X. Ma , Pest Manag. Sci. 2017, 73, 2063–2070. 28296068 10.1002/ps.4572

[9] C. Zhang , X. Yi , C. Chen , D. Tian , H. Liu , L. Xie , X. Zhu , M. Huang , G. G. Ying , Environ. Int. 2020, 139, 105719. 32283356 10.1016/j.envint.2020.105719

[10] M. B. Isman , Annu. Rev. Entomol. 2020, 65, 233–249. 31594414 10.1146/annurev-ento-011019-025010

[11] D. F. Borges , E. A. Lopes , A. R. Fialho Moraes , M. S. Soares , L. E. Visôtto , C. R. Oliveira , V. M. Moreira Valente , Crop Protection 2018, 110, 135–140.

[12] M. M. Cascaes , G. M. S. P. Guilhon , E. H. de Aguiar Andrade , Int. J Mol. Sci. 2015, 16, 23881–23904. 26473832 10.3390/ijms161023881 PMC4632730

[13] M. Afrokh , S. Tahrouch , Chem. Data Collect. 2023, 43, 100984.

[14] E. San Miguel , Econ. Bot. 2003, 57, 231–244.

[15] O. Hnatyszyn , P. Arenas , A. R. Moreno , R. Rondina , J. D. Coussio , Rev. Soc. Parag. 1974, 14, 23–57.

[16] C. C. Chen , Y. L. Huang , F. I. Huang , C. W. Wang , J. C. Ou , J. Nat. Prod. 2001, 64, 990–992. 11473445 10.1021/np000582y

[17] V. Hammiche , K. Maiza , J. Ethnopharmacol. 2006, 105, 358–367. 16414225 10.1016/j.jep.2005.11.028

[18] A. Drioiche , S. Amine , J. Essential Oil-Bearing Plants 2020, 23, 902–917.

[19] G. L. B. Castagnino , Pesqui. Agropecu. Bras. 2012, 47, 738–744. 10.1590/S0100-204X2012000900004 PMC3863766 24353353

[20] S. Wang , S. C. Li , F. S. Cheng , T. Ren , D. H. Fan-Li , Chem. Biodivers. 2022, 19, e202200351. 36053146 10.1002/cbdv.202200351

[21] L. Nahar , H. R. El-Seedi , S. A. M. Khalifa , M. Mohammadhosseini , S. D. Sarker , Molecules 2021, 26, 4766. 34443352 10.3390/molecules26164766 PMC8400350

[22] H. Zerrad , H. Bakrim , M. Moullamri , M. Bakkali , F. Alibrando , F. Cacciola , M. Mondello , P. Dugo , L. Mondello , A. Arakrak , A. Laglaoui , Oil Res. 2024, 36, 173–184.

[23] F. Haddouchi , T. M. Chaouche , Y. Zaouali , R. Ksouri , A. Attou , A. Benmansour , Chem. 2013, 141, 253–258. 10.1016/j.foodchem.2013.03.007 23768355

[24] J. M. Deguenon , J. Zhu , S. Denning , M. H. Reiskind , D. W. Watson , R. Michael Roe , J Med. Entomol. 2019, 56, 1704–1714. 31237324 10.1093/jme/tjz107

[25] Ľ. Cagáň , M. Apacsová Fusková , D. Hlávková , O. Skoková Habuštová , Plants 2022, 11, 3077. 36432806 10.3390/plants11223077 PMC9692832

[26] N. Ntalli , A. Skourti , E. P. Nika , M. C. Boukouvala , N. G. Kavallieratos , Environ. Sci. Poll. Res. 2021, 28, 42763–42775. 10.1007/s11356-021-13592-4 33825104

[27] M. Najem, M. Bammou, L. Bachiri, E. H. Bouiamrine, J. Ibijbijen, L. Nassiri, *Evidence-based Complementary and Alternative Medicine* **2020**, *1*, 5739786. 10.1155/2020/5739786 PMC7474356 32908563

[28] S. B. Chaaban , S. H. Hamdi , K. Mahjoubi , J. M. B. Jemâa , J. Plant Diseases Protec. 2019, 126, 237–246.

[29] N. G. Kavallieratos , E. P. Nika , A. Skourti , N. Ntalli , M. C. Boukouvala , C. T. Ntalaka , F. Maggi , R. Rakotosaona , M. Cespi , D. R. Perinelli , A. Canale , G. Bonacucina , G. Benelli , Molecules 2021, 26, 1812. 33806970 10.3390/molecules26061812 PMC8004781

[30] Z. Hou, X. Wang, H. Wu, Y. An, J. Zhang, J. Wang, J. Liang, *Journal of the Chin. Cereals Oils Assoc*. **2022**, *37*, 7–12.

[31] S. Ikawati , T. Himawan , A. L. Abadi , H. Tarno , Biodiversitas 2020, 21, 4301–4308.

[32] A. Plata-Rueda , J. C. Zanuncio , J. E. Serrão , L. C. Martínez , Plants 2021, 10, 2513. 34834876 10.3390/plants10112513 PMC8622527

[33] B. A. Ayil-Gutiérrez , J. M. Villegas-Mendoza , Z. Santes-Hernández , A. D. Paz-González , M. Mireles-Martínez , N. M. Rosas-García , G. Rivera , Nat. Prod. Commun. 2015, 10, 1955–1958. 26749835

[34] J. Lazarević , I. Kostić , D. Šešlija Jovanović , D. Ćalić , S. Milanović , M. Kostić , Plants 2022, 11, 3587. 36559699 10.3390/plants11243587 PMC9783734

[35] M. Fusková , Ľ. Cagáň , J. Central Euro. Agri. 2021, 22, 443–449.

[36] J. Tampe , L. Parra , K. Huaiquil , A. Quiroz , J Soil Sci Plant Nutr 2016, 16, 48–59.

[37] A. G. W. U. Perera , M. M. S. C. Karunaratne , S. D. M. Chinthaka , Res 2022, 97, 101976.

[38] S. Gao , M. Guo , Y. Yin , X. Zhang , Y. Zhang , K. Zhang , Science 2023, 58, 355–369.

[39] A. La Pergola , C. Restuccia , E. Napoli , S. Bella , S. Brighina , A. Russo , P. Suma , J. Essential Oil Res. 2017, 29, 451–460.

[40] Y. Huang , S. L. Lam , S. H. Ho , J Stored Prod Res 2000, 36, 107–117.

[41] Y. S. Xie, R. P. Bodnaryk, P. G. Fields, *Canadian Entomologist* **1996**, *128*, 865–875.

[42] R. R. Farrar , J. D. Barbour , G. G. Kennedy , Ann. Entomol. Soc. Am 1989, 82, 593–598.

[43] S. Manuwoto , J. Mark Scriber , J Econ Entomol 1982, 75, 163–167.

[44] K. Rharrabe , E. Jacquin-Joly , F. Marion-Poll , Ecol Evol 2014, 2, 5.

[45] G. Jilani , R. C. Saxena , J Econ Entomol. 1990, 83, 629–634.

[46] L. L. McDonald, R. H. Guy, R. D. Speirs,‘,Preliminary Evaluation of New Candidate Materials as Toxicants, Repellents, and Attractants against Stored-Product Insects, US Agricultural Research Service, **1970**. (No. 882).

